# Construction of in vitro liver-on-a-chip models and application progress

**DOI:** 10.1186/s12938-024-01226-y

**Published:** 2024-03-15

**Authors:** Jie Liu, Yimei Du, Xinxin Xiao, Daopeng Tan, Yuqi He, Lin Qin

**Affiliations:** 1 Guizhou Engineering Research Center of Industrial Key-Technology for Dendrobium Nobile, School of Pharmacy, Zunyi Medical University, Zunyi, 563000 China; 2 Joint International Research Laboratory of Ethnomedicine of Ministry of Education, Zunyi Medical University, Zunyi, 563000 Guizhou China; 3 The Second Affiliated Hospital of Zunyi Medical University, Zunyi, 563000 China

**Keywords:** Liver, Liver-on-a-chip, Hepatotoxicity tests, Disease modeling, Drug screening, Chip combinations

## Abstract

The liver is the largest internal organ of the human body. It has a complex structure and function and plays a vital role in drug metabolism. In recent decades, extensive research has aimed to develop in vitro models that can simulate liver function to demonstrate changes in the physiological and pathological environment of the liver. Animal models and in vitro cell models are common, but the data obtained from animal models lack relevance when applied to humans, while cell models have limited predictive ability for metabolism and toxicity in humans. Recent advancements in tissue engineering, biomaterials, chip technology, and 3D bioprinting have provided opportunities for further research in in vitro models. Among them, liver-on-a-Chip (LOC) technology has made significant achievements in reproducing the in vivo behavior, physiological microenvironment, and metabolism of cells and organs. In this review, we discuss the development of LOC and its research progress in liver diseases, hepatotoxicity tests, and drug screening, as well as chip combinations. First, we review the structure and the physiological function of the liver. Then, we introduce the LOC technology, including general concepts, preparation materials, and methods. Finally, we review the application of LOC in disease modeling, hepatotoxicity tests, drug screening, and chip combinations, as well as the future challenges and directions of LOC.

## Introduction

The liver, which is the largest internal organ in the human body, plays a crucial role in drug metabolism. The basic unit of the liver is the hepatic lobule, which is hexagonal [1]. Hepatic lobules serve as the fundamental structural and functional units for liver synthesis, decomposition, transformation, and storage. Hepatic lobules are composed of central veins, liver plates, and hepatic sinusoids. The central vein, located at the center of the hepatic lobule, is surrounded by hepatocytes and hepatic sinusoids. It serves as an important blood vessel for material exchange between the liver and other organs. Hepatocytes are found in the sinus space and are arranged in hepatic plates around the central vein. The hepatic sinusoid is situated on the hepatic plate, and blood flows through it to reach the central vein. Bile ducts are formed by local depressions in adjacent liver cells. The bile secreted by liver cells is discharged into the common hepatic duct through the bile duct. The portal area is located at the edge of the hepatic lobules, including interlobular veins, interlobular arteries, and interlobular bile ducts.

**Fig. 1:**
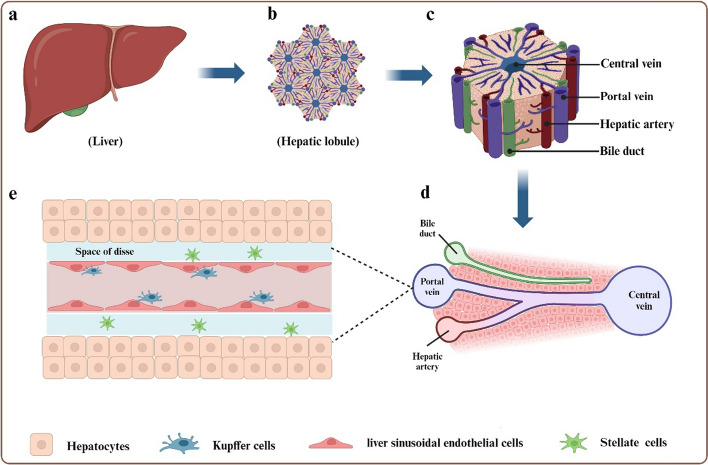
Schematics of liver structure. **a** Liver. **b**, **c** A schematic image of hepatic lobules and Internal structure. **d** Simple cross section of hepatic lobules. **e** Various types of cells and distribution of hepatic lobules. Including HCs, LESCs, HSCs and KCs. This figure was adapted and reproduced based on reference [6] using BioRender (agreement number: YW26FJ9BNL)

The liver is composed of two main types of cells: parenchymal cells (PCs), specifically hepatocytes (HCs), and non-parenchymal cells (NPCs), including liver sinusoidal endothelial cells (LSECs), hepatic stellate cells (HSCs), Kupffer cells (KCs), and bile duct cells. PCs make up ~ 80% of liver cells, while NPCs account for the remaining 20% [2]. Hepatocytes play a significant role in metabolism, detoxification, and bile acid synthesis. LSECs [3] are arranged within the low shear and sinusoidal capillary channels of the liver, making them the most abundant non-parenchymal liver cell population. The fenestra is a unique morphological feature of LSECs, forming a permeable barrier and participating in the exchange between liver cells and sinusoidal blood. HSCs [4] are mesenchymal cells found in the Disse space. After activation, they transform into myofibroblasts, which deposit collagen, leading to fibrosis and cirrhosis. KCs [5] are resident macrophages located in the sinusoidal space of the liver and participate in immune system responses. See also Fig. 1.

**Fig. 2:**
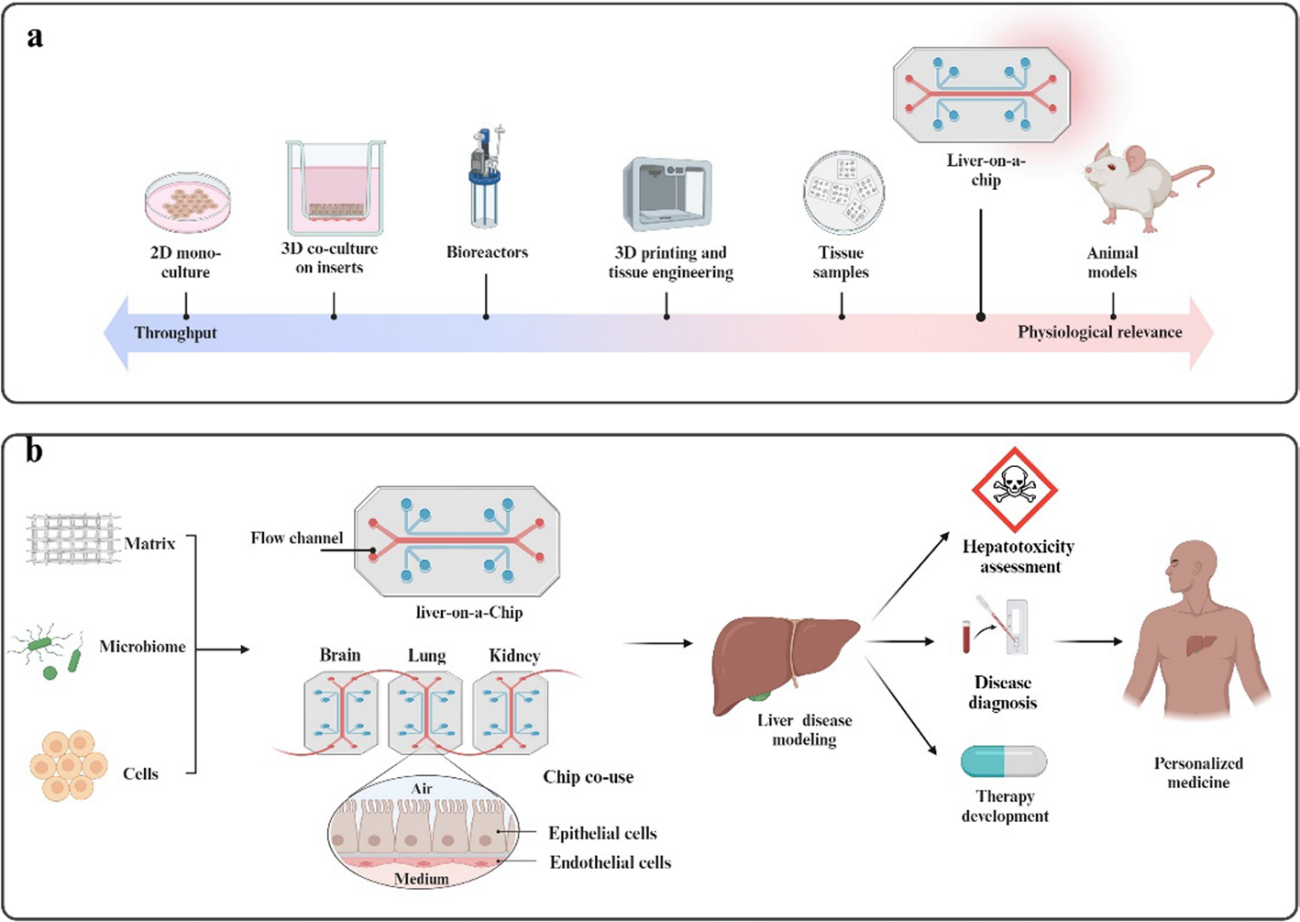
Schematics of the development and application of LOC. **a** Schematic representation of the current in vitro liver tissue culture and engineering platform and in vivo model. **b** Schematics of applications of LOC. LOC can be used for liver toxicity, disease diagnosis, and drug development. LOC can be combined with other chips for comprehensive research. This figure was adapted and reproduced based on reference [1] using BioRender (agreement number: BD26FJATKX)

An Organ-on-a-Chip (OOC) is a microfluidic device that contains bioengineered tissue with or without a portion of natural tissue, which can simulate key structures and functions in the body in vitro. Liver-on-a-Chip (LOC) is a chip technology based on microfluidics and in vitro cell culture. It simulates the liver microenvironment in vitro by controlling microfluidics, mechanical forces, and biochemical indicators. LOC is commonly used to evaluate liver function, for example, for functional simulation of liver partition structure characteristics, hepatotoxicity tests, and metabolic capacity studies. According to their distinct functions, LOC can be divided into chips used for disease diagnosis, hepatotoxicity tests, drug research and development, drug metabolism research, multichip combinations, and other functions. See also Fig. 2.

## Materials and preparation methods of liver-on-a-chip

### Composition materials of liver-on-a-chip

The basic materials of LOC include synthetic polymers and hydrogels. Synthetic polymers have the characteristics of being lightweight, low-cost, optically transparent, and chemically resistant. The most commonly used synthetic polymer is polydimethylsiloxane (PDMS) [7], which is easy to use in soft lithography, optically transparent, highly elastic, gas-permeable, biocompatible, and suitable for long-term culture of cells in a closed chamber. However, PDMS also has drawbacks [8, 9], such as poor hydrophobicity, absorbance, and a long preparation process, making it difficult to produce on a large scale. Similar materials to PDMS are polymethylmethacrylate (PMMA) and poly(*N*,*N*-dimethylacrylamide) (PDMA) hydrophilic polymers. With the development of chip technology, thermoplastic polymers, such as polycarbonate (PC), polymethyl methacrylate (PMMA), polystyrene (PS), and cycloolefin polymers (COPs) [10], especially cycloolefin copolymers (COCs), have become materials for large-scale manufacturing of low-cost microfluidic devices. COC is an engineering thermoplastic formed by copolymerization of cyclic monomers, such as norbornene and ethylene. COC has excellent optical properties, low autofluorescence, good transparency, and good biocompatibility. Moreover, it has the characteristics of no absorption or low absorption of chemicals, it has been approved by the Food and Drug Administration [8]. But COC also has its drawbacks, due to its impermeability to oxygen. At present, alternative elastomers have been adopted, such as polyester elastomers, tetrafluoroethylene propylene elastomers, and thermoplastic elastomers. Alternative elastomers are expected to become the main skeleton materials for LOC in future.

Another commonly used material is hydrogel. Hydrogels have high biocompatibility, permeability, and stiffness. They also enable the exchange of substances between cells and the extracellular matrix (ECM). In addition, hydrogels can simulate the natural microenvironment by adjusting their biochemical and physical properties to change cell behavior, such as adhesion, proliferation, migration, and differentiation. Hydrogels can be divided into natural, synthetic, and hybrid hydrogels according to their composition [11]. Natural hydrogels include polysaccharides and proteins. Their chemical composite materials and fiber structure are like those of natural ECM [12]. Synthetic hydrogels are biologically inert materials suitable for cell culture [13]. Common synthetic polymers include polyethylene glycol (PEG), polylactic acid (PLA), polyvinyl alcohol (PVA), and polyacrylic acid (PAA). Hybrid hydrogel is prepared by mixing natural hydrogel and synthetic hydrogel [14].

### Auxiliary controls for liver-on-a-chip

LOCs usually require an appropriate flow rate, pH, and temperature. These parameters depend on many auxiliary controls for regulation. Auxiliary controls include biosensors, micropumps, scaffolds, bionic membranes, and electrodes. Biosensors [6] are designed to overcome the limitations of LOC in detection and characterization. Biosensors can be integrated into LOCs to monitor oxygen levels, temperature, barrier integrity, biomarkers, evaluate availability, and reliability. Diverse types of biosensors are installed according to chip monitoring needs to meet experimental needs. Micropumps can deliver culture media to cells at optimal flow rates. Most traditional micropumps include valves, diaphragms, and piezoelectric components. There are also some valveless micropumps that can be used in the fields of microfluidic systems. These micropumps can supply nutrients and remove waste in microfluidic devices for cell culture and tissue engineering [15]. Biomimetic membranes are barrier membranes in chip systems. LOCs usually use commercial porous polymer membranes [16] and patterned polymer membranes for cell culture. Moreover, natural basement membranes and Micro/Nanoporous membranes have good physical and metabolic properties, and are commonly used in LOC [17, 18].

### Preparation of LOC

**Fig. 3:**
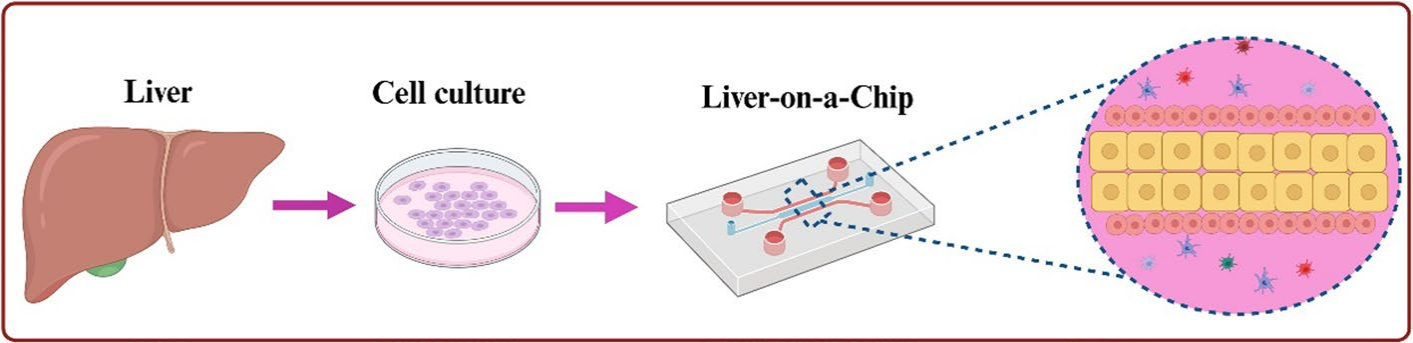
Preparation process and internal structure of LOC. First, extract target cells in vitro for cultivation. Then prepare the LOC motherboard. Finally, integrate cells, microfluidics, and auxiliary controls into LOC. This figure was adapted and reproduced based on reference [2] using BioRender (agreement number: EE26FJBHFK)

The preparation of LOC involves six steps: the first step is pattern production, for example, using CAD software to create the required patterns. The second step is to create an expert template on the chip. It can be selected using either mask lithography or maskless lithography methods. Femtosecond laser-based selective laser etching (SLE) and laser welding techniques also can be used for making the template [19]. The third step is to create a LOC. The main methods for preparing LOCs include soft lithography, 3D bioprinting, and micropatterns. Soft lithography is the most widely used technology. It is replicated by the micromachining expert produced by the lithography method, and the elastic polymer is micro-formed to generate patterns. With soft lithography, PDMS can be sealed into another PDMS or other surface in a reversible or irreversible manner to design a multilevel chip [20]. 3D bioprinting is a new technology that integrates biomaterials into 3D printing to achieve fine spatial control and form a physiologically relevant 3D tissue structure [21]. 3D bioprinting can be divided into stereolithography, extrusion, and laser-assisted bioprinting. However, it is limited by the printing resolution. Recent research suggests that micropattern technology has good development prospects and can be used as an alternative method for LOC manufacturing [22]. The fourth step is the plasma bonding of chip materials. The fifth step is equipment testing. Finally, the last step consists of LOC equipment evaluation and testing and surface treatment of cell culture materials [23]. See also Fig. 3.

### Application of LOC

#### LOC based on liver structure, zoning, and cell type

The structure and the zoning in the liver exhibit different functions and physiological effects. Ghafoory et al. developed a liver acinar device that generates hypoxia gradients in vitro [24]. It connects four chips (each chip containing two chambers) in series to obtain eight interconnected chambers and inoculates HepG2 (Human Hepatocellular Carcinoma Cells) cells. The oxygen consumption of HepG2 cells under the condition of flowing medium establishes a gradient from normal oxygen to hypoxia in the chamber. The chip can use for in vitro analysis of cells in a normoxic to hypoxic gradient. Liu et al. developed a three-vessel LOC (TVLOC) based on double-layer microspheres for reconstructing tissue-to-tissue interfaces [25]. It includes the construction of hepatic lobular cell culture zones in vitro through the hepatic artery, portal vein, and central vein. HCs and HSCs are encapsulated in bilayer microspheres to form tissue-to-tissue interface. LSECs form a vascular network around the bilayer microspheres to produce vascularized liver microstructures. The chip reconstructs hepatic lobules in vitro and provides a concentration gradient for cells to simulate the microenvironment in vivo. It can also predict drug hepatotoxicity, drug metabolism, and in vitro tumor invasion mechanisms.

Vascular liver chips have become a method for simulating and creating complex 3D tumor microenvironment (TME) structures. Özkan et al. created a 3D vascularized hepatocellular carcinoma (HCC) on chips (HCCoCs) composed of HCC cells, LSECs, HSCs, and KCs, which can simulate the liver hardness under normal or cirrhosis conditions [26]. HCCoCs can be used to investigate how the TME, and drug delivery methods can regulate HCC chemoresistance, vascular permeability, and solute transport. Lee et al. constructed a 3D tumor sphere invasion detection liver chip to monitor the fusion and invasion potential of HepG2 cells and human mesenchymal stem cells (hMSCs), as well as HSCs and HepG2 cells. This chip is a model for detecting the fusion and invasion mechanisms of HepG2 with hMSC and HSC cells [27]. Kuang et al. established a hypoxia 3D chip to study the behavior of HSCs and the TME. By simulating different types of TME, the migration behavior of HSCs under different conditions and the effect of TGF-β1 on their migration can be observed [28]. See also Table 1.

**Table 1:** LOC established based on liver structure, zoning, and cell type

Picture	Cell type	Description	References
	HepG2	Four chips (each chip containing two chambers) are connected in series to form eight interconnected chambers. Hypoxia gradient is produced in vitro, simulating the hepatic acinar. For in vitro analysis in normoxic to hypoxia gradient	[24]
	HCs、HSCs、LSECs	A three-vessel liver chip composed of hepatic artery, portal vein, and central vein is used to reconstruct hepatic lobules in vitro. To drug hepatotoxicity, metabolism, and in vitro tumor invasion mechanisms	[25]
	HSCs、KCs、LSECs	Simulate the liver hardness of normal or cirrhosis, revealing the vascular properties of TME during cancer and the tumor's response to drugs	[26]
	HSCs、 hMSCs、HepG2	A model for detecting the fusion and invasion mechanisms of HepG2 with hMSCs and HSCs cells in TME	[27]
	HSCs	To study the behavior and TME of HSCs in liver tumor matrix and observe the migration behavior of HSCs under different conditions and the effect of TGF-β1 on its migration	[28]

The figures in this table were created with BioRender.com (agreement number: YH26HQ2HTX)

#### LOC for disease modeling and drug screening

Liver cancer is a serious liver disease. Currently, there are few in vitro models available for liver cancer research. Kulsharova et al. utilized Huh-7 liver cancer cells to develop liver chips for liver cancer detection [8]. This chip is prepared by mixing COC and PDMS, and Huh-7 liver cancer cells are cultured under dynamic conditions. Non-alcoholic fatty liver disease (NAFLD) is a common chronic liver disease that can lead to liver steatosis, cirrhosis, cancer, and cardiovascular disease. Wang et al. studied an NAFLD liver chip model based on human-induced pluripotent stem cells (hiPSCs)) [29]. The chip can characterize the pathological features of NAFLD in the liver through free fatty acids (FFAs). After FFA induction, liver lipid droplets and triglycerides form, and the expression of genes related to lipid metabolism is upregulated. This indicates the successful establishment of the NAFLD model. Du et al. established a biomimetic liver lobule chip (LC) with more accurate on-chip NAFLD. This chip model allows for the observation of changes in lipid distribution in hepatic lobules as NAFLD progresses [30].

Liver-related drug metabolism is an essential aspect of pharmacokinetic toxicity research. Fanizza et al. developed a liver chip based on human-induced pluripotent stem cells (iPSCs) [31]. It interconnects iPSC-derived liver cells (iHep) and endothelial cells (iEndo) for cultivation to simulate the structure of liver sinuses. It is used for the evaluation of donepezil as a drug for Alzheimer's disease and liver toxicity screening. Chen et al. designed a liver chip to study the anti-cancer effects of statins and their metabolites [32]. The results showed that the active metabolites of simvastatin significantly reduced the activity of human prostate cancer and liver cancer cells. It also demonstrates no significant toxicity to normal cells, such as primary liver cells and fibroblasts. See also Table 2.

**Table 2:** LOC for disease modeling and drug screening

Picture	Cell type	Description	References
	Huh-7	It combines COC with PDMS and can be used for screening and cell analysis of lipid intermediates. It is also suitable for rapid prototyping and cultivate functional liver cancer Huh-7 cell lines	[7]
	hiPSCs	This chip combines stem cell organoids with organ chip technology and is established using a hiPSC-derived liver organoid chip system	[29]
	HepaRG^a^ LX2 cells^b^	For the detection of NAFLD. The changes of lipid partition in hepatic lobules with the progress of NAFLD were proved at chip level	[30]
	iHep, iEndo	It was prepared using the innovative equipment MINERVA 2.0. Evaluation of the drug donepezil for Alzheimer 's disease and screening for hepatotoxicity	[31]
	HepG2	It is used to study the anti-cancer effects of statins and their metabolites	[32]

^a^HepaRG: hepatoma cells

^b^LX2 cells: Human hepatic stellate cells. The figures in this table were created with BioRender.com (agreement number: OK26HQ3492)

#### LOC for hepatotoxicity study

Drug-induced hepatotoxicity or liver injury (DILI) is the main reason for discontinuing clinical trials and drug withdrawal from the market [25]. Kwon et al. conducted a study on a liver acinar dynamics chip (LADY) that can replicate half of the liver acinar diamond shape to induce liver zoning [33]. The 3D co-culture of HCs and LSECs can accurately detect the drug-induced zonal hepatotoxicity and improve the resistance to acetaminophen toxicity. Additionally, the chip successfully forms a metabolic zone through the medium flow that corresponds to the blood flow in the body. The chip is mainly used for hepatotoxicity research. Studies have shown that 3D in vitro liver models with co-cultured liver PCs and NPCs can better predict hepatotoxicity than models with only HCs. The addition of NPCs promotes the inflammatory components of DILI endothelial channels. Janani et al. utilized 3D bioprinting technology to develop an in vitro human vascularized liver chip for liver toxicity testing. It simulates the in vitro hepatic lobular structure using PCs and NPCs [34]. This chip adopts human adipose mesenchymal stem cell-derived hepatocyte-like cells (HLCs), and human umbilical vein endothelial cells (HUVECs), and human hepatic stellate cells (HHSCs). It is mainly used for measuring DILI and its application in drug toxicity and drug screening. Zheng et al. studied a 3D multicellular liver chip (3DDMLoC) which can be used for in vitro hepatotoxicity screening. It was developed to reproduce the microenvironment of in vivo liver tissue, including the simulation of hepatic sinusoid, peri-sinusoidal space and continuous liquid perfusion [35]. Li et al. developed a 3D liver chip for the detection of liver toxicity caused by nanoparticles, with a primary focus on studying the potential liver toxicity of superparamagnetic iron oxide nanoparticles (SPIONs) under microfluidic conditions [36]. This chip simulates the in vivo environment of the liver, the cell culture microenvironment, and the interactions between cells. Also, it can screen for drug-induced partitioned hepatotoxicity. Wu et al. developed a digital chip for hepatotoxicity assessment and anti-hepatoma immunotherapy based on extracellular vesicles by combining microporous arrays and cell microspheres [37]. This chip contains cell microspheres composed of HepG2 cells, HUVECs, and human foreskin fibroblasts (HFF-1), which can accurately represent the in vivo situation. It also includes over one hundred organisms, transforming traditional holistic analysis methods into digital analysis methods, not only improving the parallelism of analysis units but also reducing the variability of fluorescence detection analysis. See also Table 3.

**Table 3:** LOC for hepatotoxicity study

Picture	Cell type	Description	References
	HCs, LSECs	Simulate the structure of liver acini and induce liver partitioning. Improve resistance to acetaminophen toxicity for liver toxicity testing	[33]
	HLCs, HUVECs, HHSCs	Use squeeze bioprinting technology to establish for DILI liver toxicity testing	[34]
	HUVEC, HepaRG^a^	Simulate hepatic sinus, use 3D dynamic multi-cell co-culture for in vitro hepatotoxicity screening	[35]
	HCs	Use for liver toxicity detection of nanoparticle SPION and screen for drug-induced partitioned liver toxicity	[36]
	HepG2, HUVECs, HFF-1	Use chip technology, microporous arrays, and cell microspheres. Uniform microspheres and microporous arrays divide the traditional “single pot” analysis unit into hundreds of independent analysis sheets	[37]

^a^HepaRG: hepatoma cells

The figures in this table were created with BioRender.com (agreement number: FU26HQ3R6S)

### Chip combinations

#### Intestine–liver chip

Enterohepatic circulation refers to the phenomenon where drugs discharged into the intestine through bile are reabsorbed in the intestine and returned to the liver through the portal vein. Intestine–liver chips can be used to predict the effects of oral drug administration and in vitro first pass metabolism. De Gregorio et al. constructed a 3D in vitro intestine–liver chip to simulate the first pass mechanism in vivo [38]. The chip simulated the first pass metabolism of ethanol and revealed the protective effect of the intestine on liver injury. Kang et al. developed an intestine–liver chip in which cell viability and liver function were significantly enhanced after microbial-derived small molecule treatment [39]. Moreover, the extracellular vehicles (EVs) of the microbial community were isolated. EVs can affect adjacent cells near secretory cells, thereby exerting anti-inflammatory, anti-apoptotic, and immunosuppressive effects to promote tissue repair. One of the challenges in drug development is predicting human pharmacokinetics. The micro-physiological system (MPS) composed of multiple organ chips may provide more accurate expression of in vitro drug metabolism, efficacy, and toxicity. Milani et al. studied the application of an intestine–liver chip in the quantitative pharmacokinetic study of mycophenolate mofetil in vitro [40]. The purpose is to explore and verify the use of intestine–liver MPSs in ADME applications. The gut–liver axis (GLA) is especially important for maintaining human health. Yang et al. developed a GLA-MPS chip platform that can culture intestinal cells and hepatocytes and apply different perfusion flows to improve cell viability and function to form barrier tissues [41]. The platform mimics the physiological blood perfusion and circulation of GLA in vivo. It can also achieve independent physiological flow perfusion of each organ in the cell incubator. GLA-MPS can be widely used in disease modeling related to inter-tissue interactions, such as fatty liver disease. See also Table 4.

**Table 4:** Intestine–liver chip

Picture	Cell type	Description	References
	Caco-2^a^, HepG2	Predict oral drug administration and in vitro first pass metabolism, simulate ethanol first pass metabolism	[38]
	HepG2	Study the EVs of microbiota and the interaction between microbiota and host cells	[39]
	Caco-2^a^, HCs, HT29^b^	Study the PK of mycophenolate ester and its two main metabolites	[40]
	Caco-2, HepG2	Consist of four microfluidic structures (cell culture layer, porous membrane, circulation layer, and control layer). Allow independent physiological perfusion of each organ in the cell culture chamber for disease modeling	[41]

^a^Caco-2: Human intestinal epithelial cells

^b^HT29: HT29 human colorectal adenocarcinoma cells. The figures in this table were created with BioRender.com (agreement number: KJ26HQ45N8)

#### Other combinations

Chip combinations can comprehensively reflect the interaction between various organs in the body and the underlying mechanism. Nguyen et al. designed a multi-organ chip based on human kidney and liver organoids to investigate the therapeutic efficacy and biological distribution of EVs derived from mesenchymal stromal cells (MSCs) [42]. This chip can be used to study the regenerative potential and organ distribution of MSC-EVs in acute kidney injury models. Zandi Shafagh et al. developed a multifunctional liver–pancreas chip with pneumatic drive, which can identify the metabolic response characteristics of prediabetic hyperglycemia in humans and can transfer mass between different tissue models at a specific flow rate [43]. Koenig et al. designed a liver-brain chip for evaluating drug blood–brain barrier permeability [44]. This chip combines the blood–brain barrier model derived from hiPSCs with cortical brain and hepatic sphere models and evaluates metabolism with atenolol and propranolol. Both substances exhibit osmotic behavior like in vivo. The chip can measure the concentration of parent compounds and the distribution of metabolites at the blood–brain barrier. Madiedo et al. developed a liver-lung chip for inhalation toxicity assessment [45]. The chip can simulate active and functional in vitro interference in a stress environment for inhalation toxicity assessment. See also Table 5.

**Table 5:** LOC combined with other chips

Picture	Cell type	Description	References
	MSCs	Liver–kidney chip. To study the therapeutic effect and bio-distribution of MSC-EVs	[42]
	PHH^a^	Liver–pancreas chip. Identify the characteristics of human metabolic response to pre-diabetes hyperglycemia	[43]
	hiPSCs	Liver–brain chip. Consist of human blood–brain barrier, cortical brain, and liver models derived from fully autologous iPSC. Evaluate the permeability of drug blood–brain barrier	[44]
	Calu-3^b^, HepG2/C3A^c^	Liver–lung chip. Active and functional in vitro interference can be simulated in a stress-induced environment for inhalation toxicity assessment	[45]

^a^PHH: primary human hepatocytes

^b^Calu-3: Human lung Adenocarcinoma Cells

^c^HepG2/C3A: C3A hepatoma cells. The figures in this table were created with BioRender.com (agreement number: RV26HQ576H)

## Conclusion

The liver is an important organ that performs multiple functions and is influenced by numerous factors. DILI and liver diseases can lead to liver dysfunction and damage to basic liver activity [46]. However, over the past few decades, there has been a lack of comprehensive liver disease research due to the lack of suitable in vivo or in vitro liver models. The commonly used in vitro cell models and animal models also have certain limitations. LOC is an engineered microenvironment system in which several types of cells can be co-cultured. It can be used to simulate the liver microenvironment, zoning, the oxygen gradient, and liver metabolic capacity. LOC has been successfully applied in liver toxicity research, disease modeling, and drug development, and it has the potential to improve our understanding of clinical applications and personalized medical research. Additionally, LOC is a powerful preclinical research tool that is expected to effectively alleviate the growing ethical issues of animal experimentation.

However, LOC also has its limitations. For instance, there is a need for better preparation materials and methods in constructing LOCs to reduce preparation time and cost, as well as to enhance the accuracy of the LOCs. The preparation process of chips and the optimization and selection of preparation materials are areas that need to be explored in LOC development. ECM plays a crucial role as a biological tissue material in LOC preparation, and ECM materials with different compositions are essential for the success of LOCs. Embedding suitable ECM into MPS can facilitate the accurate establishment of liver in vitro MPS [47]. Furthermore, different cell culture techniques are key factors in LOC. The dynamic 3D sphere cell culture technology, when embedded in MPS, can overcome the issue of short survival time in traditional chip cell culture [48]. Additionally, by incorporating specific biosensors into MPS, continuous monitoring of in vitro drug toxicity can be achieved [49]. Moreover, it is possible to connect the liver hypoxia chip with the albumin monitoring system to develop an albumin detection system with a liver hypoxia chip for assessing liver function [50]. Furthermore, liver chip technology can be combined with mathematical modeling and simulation techniques to study drug metabolism [51], as well as to evaluate the efficiency of lineage reprogramming in tumor cells [52].

Furthermore, the currently cultured cells are limited to in vitro conditions. However, if there are more human cell samples available, it would be possible to better predict human-specific clinical disease outcomes. Currently, human cells, such as HepG2, HepaRG, PHH, and hiPSC, are used in LOC. hiPSC, in particular, has shown great potential as a source of mature liver cells [53, 54]. However, the technology in this area is still imperfect, and there is a need to improve the cultivation conditions of hiPSC. KCs play a role in immune system responses [5]. LSECs are specialized endothelial cells with clearance and immune functions [55]. Bile duct cells are primarily involved in biliary-related diseases and the regulation of bile acids in the body [56]. However, there are currently limited in vitro models targeting NPC (non-parenchymal) cells, such as KCs, LSECs, and bile duct cells, which fail to effectively reflect liver changes in vitro. Therefore, the development of in vitro NPC-like liver models holds significant prospects for subsequent drug clinical research and toxicity studies.

Moreover, currently, LOC is not widely used in combination with other chips, so further research is needed in this area. The combination of chips is a growing trend in chip technology development. This article briefly discusses the combination of liver, intestine, kidney, brain, and lung chips. However, chip combinations are not limited to these types alone. There are also combinations, such as liver–pancreatic chips, liver–heart chips, and liver–skin chips [57–59]. Currently, there are few reports on the use of chip combinations, especially in the case of multi-chip combinations involving three or four chips. Additionally, there are limited reports on chip combinations for specific diseases. For example, the combination of liver and testicular chips is used for in vitro detection of male diseases [60]. The combination of liver chips, ovarian chips, and breast chips is used for the detection of female ovarian diseases [61]. Furthermore, multi-chip research faces practical challenges, such as limitations in obtaining human cell types in vitro, limited sample size, and chip compatibility. Additionally, the combination of multiple chips requires higher standards for chip biomaterials. These issues are areas that future research should focus on. The combination of multiple chips is a key area for future development, and there are increasing challenges that need to be addressed. LOC combination models can reflect in vivo processes in an effective way.

## Data Availability

Not applicable.

## Abbreviations

LOC: Liver-on-a-chip
OOC: Organ-on-a-chip
PCs: Parenchymal cells
HCs: Hepatocytes
NPCs: Non-parenchymal cells
LSECs: Liver sinusoidal endothelial cells
HSCs: Hepatic stellate cells
KCs: Kupffer cells
PDMS: Polydimethylsiloxane
PMMA: Polymethylmethacrylate
PDMA: Poly(*N*,*N*-dimethylacrylamide)
PC: Polycarbonate
PS: Polystyrene
COPs: Cycloolefin polymers
COCs: Cycloolefin copolymers
ECM: Extracellular matrix
PEG: Polyethylene glycol
PLA: Polylactic acid
PVA: Polyvinyl alcohol
PAA: Polyacrylic acid
SLE: Selective laser etching
TME: Tumor microenvironment
HCC: Hepatocellular carcinoma
hMSCs: Human mesenchymal stem cells
NAFLD: Non-alcoholic fatty liver disease
hiPSCs: Human-induced pluripotent stem cells
FFAs: Free fatty acids
iHep: IPSC-derived liver cells
iEndo: Endothelial cells
DILI: Drug-induced hepatotoxicity or liver injury
HLCs: Hepatocyte-like cells
HUVECs: Human umbilical vein endothelial cells
HHSCs: Human hepatic stellate cells
SPIONs: Superparamagnetic iron oxide nanoparticles
HFF-1: Human foreskin fibroblasts
MPS: Micro-physiological system
GLA: Gut–liver axis
EVs: Extracellular vesicles
MSCs: Mesenchymal stromal cells
PHH: Primary human hepatocytes
Caco-2: Human intestinal epithelial cells
HEPG2: Human hepatocellular carcinoma cells
LX2 cells: Human hepatic stellate cells
HepaRG: Hepatoma cells
HT29: HT29 Human colorectal adenocarcinoma cells
PHH: Primary human hepatocytes
HepG2/C3A: C3A Hepatoma cells
